# Why *Nutrients*?

**DOI:** 10.3390/nu1010001

**Published:** 2009-07-09

**Authors:** Peter Howe

The field of nutrition continues to attract increasing interest from health professionals, including dietitians, sports nutritionists and medical practitioners, from biomedical, agricultural, nutritional and food scientists and from health conscious consumers alike. A major driver of this increased interest is the growing global concern about health sustainability and recognition of the economic importance of developing more cost-effective strategies for health development and management which will enable us to maintain quality of life and productivity in an aging population and reduce our increasing dependence on costly medical and surgical interventions. Of the potential diet and lifestyle approaches, the concept of functional foods has generated much interest in recent years. The promise to consumers of greater opportunity for self management of their health and to food manufacturers of heightened consumer demand for healthy products has been a major impetus for innovation, marketing and regulatory change in the food industry. 

A key factor in the rapid development of functional foods has been the role of nutritional science in the identification and utilisation of bioactive nutrients. Indeed, regulatory developments such as health claims on food labels are heavily dependent on rigorous nutritional science for substantiation of claimed benefits. There has been a proliferation of new journals and information services relating to functional foods, nutraceuticals, *etc.* yet few with a direct focus on nutrients *per se*. Yet fundamental to designing, evaluating and/or promoting healthy foods is an understanding of the physiological effects of constituent nutrients, together with knowledge of their safety and efficacy, physicochemical properties, organoleptic attributes, sources, availability and sustainability, to name a few relevant fields. These and many more fields of multidisciplinary interest are encompassed in the scope of *Nutrients*. Clearly the introduction of a journal bearing the name *Nutrients* is intended to address this topic directly and in the broadest sense.

Researchers in the field of nutrition already have a wide range of journals in which to publish their findings. Many reputable, long-established journals originated through professional nutrition and dietetics organisations. Others have expanded from related disciplines to embrace nutrition. Whilst these journals vary considerably in quality and popularity, the overall impact of nutrition-related journals is relatively low compared with other fields of biomedical science. There are many possible reasons for this. One obvious one must be a relatively low readership. Yet, at the same time, there is increasing interest from professionals, industry and lay people alike in the latest developments in nutritional and functional food research. This is clearly evidenced by the recent proliferation of web-based alerting and abstracting services in the field. Moreover, the free access offered by most of these services must be raising awareness of developments in nutritional research and stimulating readership of relevant publications. We expect that *Nutrients*, as an open access online journal, will similarly impact on the field of nutrition by stimulating even greater interest in nutritional research and citation of publications, thus making an important new contribution from which existing publications may also expect to benefit.

As with other international journals in the field, *Nutrients* will receive manuscripts of original research, review articles or commentaries for expert peer review; those deemed acceptable will be published online as soon as practicable. In most cases, this will precede the formal publication date for the relevant issue. Being *open access* means that anyone can access and download journal articles directly from the website without cost. In order to do this, a fee will be charged to the contributor on acceptance of a manuscript for publication. This also means that new research can be accessed very rapidly by an unlimited readership. Moreover, we expect relevant articles in *Nutrients* to be cited by the major nutritional and food alerting services who will be able to provide free electronic links to the full text article, thus ensuring widespread awareness of newly published papers. Accordingly, we expect articles in Nutrients to be extensively cited and we hope to achieve Medline and other major journal database listings with a journal Impact Factor in a relatively short time frame. To facilitate this process, *Nutrients* will publish a variety of special issues in topical areas with invited reviews and guest editors from leaders in the field, thus ensuring interest in the new journal as well as providing a new forum for citing existing publications in the field of nutrition. Moreover, all articles will be published free of charge to the contributors in 2009. We hope that the impact of these strategies will be to stimulate heightened interest in and awareness of nutritional research. 

As stated above, the topic of *Nutrients* will be addressed in the broadest sense in this new journal. Traditional definitions and classifications of nutrients will be extended to include all food ingredients that can positively impact on health in both humans and animals. Moreover, coverage will extend from the farm through food manufacturers and consumers to the sports field or clinic. Topical issues of nutrient sources, safety and sustainability will be addressed, together with global nutritional issues ranging from under nutrition to dietary excesses and diseases of affluence. A list of suggested topics appears on the journal’s website. The list is far from exhaustive but indicates the likely emphasis of the journal’s content. Whilst its focus is clearly on nutrients and health, the journal recognises that health benefits are unlikely to be obtained from nutrients in isolation. Indeed, we anticipate that many contributions will reflect the role of nutrients and foods as an integral component of broad-based lifestyle approaches to health optimisation and even as complimentary therapies, including adjuncts to drug therapy. With this broad outline, we trust that potential contributors will be encouraged to submit papers relating to all aspects of nutrients, confident that the journal’s editorial board with its extensive multidisciplinary expertise and experience is well qualified to appraise and promote high quality research. 

Finally, I would like to congratulate the publishers, MDPI, for taking the initiative to establish a new online journal in what they had perceived to be an important field of knowledge growth and application. I trust that researchers, readers and stakeholders alike will value the investment that has been made for us by MDPI and profitably utilise our new resource.

